# Influence of increased intracranial pressure on sevoflurane-fentanyl anesthesia in major abdominal surgery

**DOI:** 10.1186/cc13609

**Published:** 2014-03-17

**Authors:** N Trembach, I Zabolotskikh

**Affiliations:** 1 Kuban State Medical University, Krasnodar, Russia

## Introduction

Increased intracranial pressure (ICP) may adversely affect sevoflurane anesthesia and the recovery period due to a disturbed cerebral blood flow. Effect of sevoflurane on cerebral hemodynamics remains controversial and depends on the extent of the initial value of ICP [1]. This study was designed to evaluate the safety of sevoflurane- fentanyl anesthesia during major abdominal surgery in patients with increased ICP.

## Methods

A total of 124 ASA 3 patients, undergoing major abdominal surgery (duration 5.6 (4.1 to 6.4) hours), were divided into two groups: with normal ICP (≤12 mmHg) (N group, 70 patients) and with ICP >12 mmHg (H group, 54 patients). Initial ICP was evaluated by venous ophthalmodynamometry [2]. ICP, mean arterial pressure (MAP) and cerebral perfusion pressure (CPP) were assessed every hour of anesthesia. Time of recovery of consciousness after anesthesia, complications and length of stay in the ICU and in the hospital were also evaluated.

## Results

Initial ICP was 8 ± 3 mmHg in the N group and 15 ± 2 mmHg in the H group. During the anesthesia ICP increased in the H group with a total increase of 33% (from 15 ± 2 to 20 ± 3 mmHg (*P *< 0.05)). In the N group ICP was stable without any significant change. Decrease of MAP after induction of anesthesia was similar in the two groups and was stable during anesthesia. CPP was stable in the N group (above 70 mmHg during the anesthesia), but in the H group CPP decreased significantly (from 82 mmHg to 63 mmHg (*P *< 0.05)). Time of recovery of consciousness in the H group was higher (32 ± 6 minutes vs. 20 ± 4 minutes (*P *< 0.05)). The incidence of postoperative delirium was higher in the H group (22.2% vs. 12.8% in the N group (*P *< 0.05)). There were no significant differences between two groups in other complications. Total length of stay in the ICU and in the hospital was higher in the H group (6 ± 2 days vs. 4 ± 2 days (*P *< 0.05) and 15 ± 3 days vs. 11 ± 2 days in N group (*P *< 0.05)).

## Conclusion

Sevoflurane-fentanyl anesthesia in patients with increased ICP was characterized by a delayed recovery with a higher incidence of postoperative delirium and higher length of stay in the ICU and in the hospital. The main cause of this is a decrease of CPP in patients with high ICP due to low craniocerebral compliance.
